# Utility of pre‐operative haemoglobin concentration to guide peri‐operative blood tests for hip and knee arthroplasty: A decision curve analysis

**DOI:** 10.1111/tme.12873

**Published:** 2022-05-11

**Authors:** Paula Dhiman, Victoria N. Gibbs, Gary S. Collins, Ben Van Calster, Gardash Bakhishli, George Grammatopoulos, Andrew J. Price, Adrian Taylor, Mike F. Murphy, Ben J. L. Kendrick, Antony J. R. Palmer

**Affiliations:** ^1^ Centre for Statistics in Medicine, Nuffield Department of Orthopaedics, Rheumatology and Musculoskeletal Sciences University of Oxford Oxford UK; ^2^ NIHR Oxford Biomedical Research Centre Oxford University Hospitals NHS Foundation Trust Oxford UK; ^3^ Nuffield Department of Orthopaedics, Rheumatology and Musculoskeletal Sciences Botnar Research Centre Oxford UK; ^4^ Nuffield Orthopaedic Centre Oxford UK; ^5^ Department of Development and Regeneration KU Leuven Leuven Belgium; ^6^ Oxford University Hospitals NHS Foundation Trust, Blood Safety and Conservation Team John Radcliffe Hospital Oxford UK; ^7^ Division of Orthopaedic Surgery The Ottawa Hospital Ottawa Ontario Canada; ^8^ NHS Blood & Transplant John Radcliffe Hospital Oxford UK; ^9^ Royal National Orthopaedic Hospital Middlesex UK

**Keywords:** blood transfusion, decision curve analysis, haemaglobin, orthopaedics

## Abstract

**Objective:**

Assess the prognostic value of pre‐operative haemoglobin concentration (Hb) for identifying patients who develop severe post‐operative anaemia or require blood transfusion following primary total hip or knee, or unicompartmental knee arthroplasty (THA, TKA, UKA).

**Background:**

Pre‐operative group and save (G&S), and post‐operative Hb measurement may be unnecessary for many patients undergoing hip and knee arthroplasty provided individuals at greatest risk of severe post‐operative anaemia can be identified.

**Methods and Materials:**

Patients undergoing THA, TKA, or UKA between 2011 and 2018 were included. Outcomes were post‐operative Hb below 70 and 80 g/L, and peri‐operative blood transfusion. Logistic regression assessed the association between pre‐operative Hb and each outcome. Decision curve analysis compared strategies for selecting patients for G&S and post‐operative Hb measurement.

**Results:**

10 015 THA, TKA and UKA procedures were performed in 8582 patients. The incidence of blood transfusion (4.5%) decreased during the study. Using procedure specific Hb thresholds to select patients for pre‐operative G&S and post‐operative Hb testing had a greater net benefit than selecting all patients, no patients, or patients with pre‐operative anaemia.

**Conclusions:**

Pre‐operative G&S and post‐operative Hb measurement may not be indicated for UKA or TKA when adopting restrictive transfusion thresholds, provided clinicians accept a 0.1% risk of patients developing severe undiagnosed post‐operative anaemia (Hb < 70 g/L). The decision to perform these blood tests for THA patients should be based on local institutional data and selection of acceptable risk thresholds.

## 1 INTRODUCTION

The number of elective primary hip and knee arthroplasties performed internationally is projected to increase exponentially for hip arthroplasty by 2050 and four‐fold for knees by 2030, with comparable projections across 20 Organisation for Economic Cooperation and Development countries.^1^, ^2^, ^3^

Hip and knee arthroplasties are highly successful procedures at improving quality of life, but the surgery can result in significant blood loss.^4^, ^5^ Blood loss can exceed 1 L during the peri‐operative period for a primary hip or knee arthroplasty procedures when accounting for visible and hidden blood losses.^6^ Despite blood conservation strategies, over 80% of patients are anaemic on discharge from hospital and up to 5% require allogenic red blood cell transfusion.^7^, ^8^, ^9^, ^10^ The recommended haemoglobin concentration (Hb) threshold for administering allogeneic red blood post‐operatively is 70 g/L, or 80 g/L in the presence of acute coronary syndrome.^11^

Routine clinical practice is to perform group and save (G&S) in all patients prior to arthroplasty surgery and a post‐operative blood test for Hb prior to discharge from hospital, however, there is limited guidance for when these investigations are indicated or can be omitted. Pre‐operative G&S and post‐operative Hb measurement may be unnecessary when the risk of requiring a blood transfusion or a patient developing severe post‐operative anaemia is acceptably low.

Previous studies have identified pre‐operative Hb thresholds that predict blood transfusion using Receiver Operator Characteristics (ROC) curve methodology, choosing pre‐operative Hb values associated with the highest combined sensitivity and specificity, and giving equal weighting to true and false positives.^12^, ^13^ However, this does not allow clinicians to consider the harm: benefit ratio they feel is appropriate for their patient cohort.

The potential benefits of a targeted approach to peri‐operative blood tests include reduced cost, improved patient experience, and shorter length of inpatient stay by preventing delays waiting for blood results and facilitating safe day‐case surgery. Potential risks of omitting these investigations include having no blood product available when required and discharging patients with severe undiagnosed anaemia. The acceptable harm: benefit ratio will differ between institutions and clinicians.

The aim of this study was to characterise the prognostic value of pre‐operative Hb for identifying patients who develop severe post‐operative anaemia or require blood transfusion following elective primary hip or knee arthroplasty. We address four research questions:

1. What are the temporal trends of post‐operative Hb below the 70 and 80 g/L transfusion thresholds, and allogeneic blood transfusion following elective primary hip and knee arthroplasty?
2. What is the relationship between pre‐operative Hb, and post‐operative Hb and blood transfusion?
3. What are the pre‐operative Hb thresholds for different risks of severe post‐operative anaemia?
4. What is the utility of different strategies using pre‐operative Hb to select patients for pre‐operative G&S and post‐operative Hb measurement?

## 2 MATERIALS AND METHODS

We performed a cohort study using electronic health records, reported in accordance to the Strengthening the Reporting of Observational Studies in Epidemiology (STROBE) and Reporting Recommendations for Tumour Marker Prognostic Studies (REMARK) statements.^14^, ^15^

### 2.1 Subjects studied

Routinely collected data from the Nuffield Orthopaedic Centre (NOC), Oxford, UK, between December 2011 and August 2018 were used. All primary hip and knee arthroplasty procedures at the NOC were included in the study, regardless of approach, implant, blood management strategy or grade of surgeon. Patients undergoing elective primary total hip arthroplasty (THA), total knee arthroplasty (TKA) or unicompartmental knee arthroplasty (UKA) were identified using the Office of Population Censuses and Surveys (OPCS) procedure codes (Classification of Interventions and Procedures, version 4) (Table S1). THA were performed using the anterolateral or posterior approach using cemented and uncemented implants. TKA were mostly performed using the medial parapatellar approach and a tourniquet. UKA were routinely performed with a minimally invasive medial arthrotomy and a tourniquet. Routine tranexamic acid administration was introduced after 2015 as intravenous with or without topical administration. Patients who had a blood transfusion within 3 months prior to surgery were excluded as they are likely to have an underlying medical condition that requires regular Hb measurement. Procedures with a missing date and duplicate entries on electronic health records were also excluded.

### 2.2 Outcomes

The primary outcome was the National Institute for Health and Care Excellence (NICE) recommended transfusion threshold of a Hb less than 70 g/L within 7 days following surgery (<70 g/L transfusion threshold: yes/no, binary).^11^

Secondary outcomes were the NICE recommended transfusion threshold for patients with acute coronary syndrome of a Hb less than 80 g/L (<80 g/L transfusion threshold: yes/no, binary)^11^; and administration of a blood transfusion intra‐ or post‐ operatively (blood transfusion: yes/no, binary) within 7 days following surgery.

All data collected between 2011 and 2018 were used for Hb measurements. Data collected between 2013 and 2018 were used for the blood transfusion analysis when electronic prescribing for blood transfusion was introduced.

### 2.3 Variables

Data were extracted for: age (years, continuous); sex (male/female, binary); pre‐operative Hb (g/L, continuous); American Society of Anesthesiologists (ASA) classification (range: 1–5, ordinal); and post‐operative Hb (g/L, continuous). The closest pre‐operative Hb value to surgery start date and time was used from within 6 months prior to surgery, and the closest value to surgery end date and time was used from within 7 days following surgery.

Pre‐operative and post‐operative Hb were categorised according to the World Health Organisation (WHO) and the International Consensus Statement (ICS) definitions of anaemia for descriptive purposes.^16^, ^17^ The WHO defines anaemia using a haemoglobin <120 g/L for females and <130 g/L haemoglobin for males; and the ICS defines anaemia using a <130 g/L for both females and males.

### 2.4 Sample size

No formal sample size calculation was carried out. The study size was limited by the number of THA, TKA, and UKA procedures undertaken at the NOC between December 2011 and August 2018.

### 2.5 Statistical analysis

#### 2.5.1 Time trends

The proportion of patients with post‐operative Hb below the 70 and 80 g/L transfusion thresholds and the proportion of patients receiving blood transfusion were described for all procedures by year of surgery.

#### 2.5.2 Regression analysis

The association between pre‐operative Hb and the outcomes was evaluated using unadjusted and adjusted logistic regression. The variables age, sex, and ASA classification were chosen a‐priori to be included in the adjusted logistic regression. We assessed non‐linearity and checked the functional form of pre‐operative Hb using fractional polynomials of degree 1.^18^ Unadjusted and adjusted odds ratios with 95% confidence intervals are presented.

#### 2.5.3 Decision curve analysis

Decision curve analysis (DCA) was used to assess and compare the utility of four intervention strategies to identify patients with post‐operative anaemia and needing a blood transfusion, and therefore, which patients should be selected for G&S prior to surgery and similarly, which patients should have post‐operative blood tests to measure their Hb.^19^, ^20^

Intervention strategies were:

1. *Test all patients*: perform pre‐operative G&S and post‐operative Hb measurement for all patients
2. *Test no patients*: do not perform pre‐operative G&S or post‐operative Hb measurement for any patients
3. *Test patients with pre‐operative anaemia*: perform pre‐operative G&S and post‐operative Hb measurement on patients with pre‐operative Hb < 130 g/L
4. *Test patients according to risk of post‐operative anaemia*: select patients for pre‐operative G&S and post‐operative Hb measurement based on risk of post‐operative anaemia (pre‐operative Hb threshold for intervention informed by unadjusted logistic regression models).

##### Risk thresholds—Clinician's preference

We considered a range of 0.1% (clinician is more concerned about undiagnosed post‐operative anaemia) to 1% (clinician is more concerned about unnecessary post‐operative blood tests) that a clinician may find acceptable for not having performed a G&S when a post‐operative blood transfusion is indicated. As the risk threshold decreases there will be more true positives (necessary G&S and post‐operative blood tests) at the expense of more false positives (unnecessary G&S and post‐operative blood tests). When a risk threshold of 0.1% (1:1000) is used, we perform a G&S and post‐operative blood test on at most 1000 patients per one true positive.

Alternatively, the odds of the risk threshold represent the maximum number of false positives that a clinician is willing to accept per true positive; known as the ‘harm to benefit ratio’. For the 0.1% threshold, the odds (harm to benefit ratio) are 1:999, hence at most 999 false positives are accepted per one true positive. Therefore, risk thresholds of 0.25%, 0.5%, 0.75% and 1% imply a clinician is willing to unnecessarily perform G&S and a post‐operative blood test on 399, 199, 133 and 99 patients to correctly identify one patient where a transfusion is required, respectively.

As the risk threshold decreases, the clinician is more averse to the risk of not having blood available for transfusion or not diagnosing severe post‐operative anaemia. The range of risk thresholds refers to the clinician's preference to perform a G&S and post‐operative blood test for a given patient, which can be discussed with the patient and thus can vary accordingly.

##### Net benefit

To assess the utility of the four intervention strategies for selecting patients for G&S and post‐operative Hb measurement we calculated net benefit, which is the net proportion of true positives, much like net profit equals revenue minus all expenditures in business.

The benefit of an intervention strategy is that is correctly identifies which patients had a post‐operative Hb < 70 g/L, <80 g/L or had a blood transfusion (and hence needed a G&S and postoperative blood test). For example, a net benefit of 0.002 is equivalent to having 2 additional patients correctly identified as needing blood transfusion per 1000 patients, without incorrectly selecting anyone who did not need a blood transfusion.

The net benefit is directly comparable to the ‘test none’ strategy (do not perform pre‐operative G&S or post‐operative Hb measurement for anyone) which by definition has a net benefit of 0 because if you never intervene you do not have any true or false positives. The difference in net benefit between all strategies can also be calculated, for example, if the net benefit increases from 0.001 when using ‘Test all: intervention for all patients’ to 0.002 when using the ‘Test patients according to risk of post‐operative anaemia’ strategy, the latter has 1 more net detected blood transfusion per 1000 patients for the same number of unnecessary G&S and post‐operative blood test carried out.

The net benefit was calculated and presented in a decision curve at risk thresholds between 0.1% and 1%, as it was deemed unlikely a clinician would risk not having blood available when the risk of needing it exceeds 1%. The DCA was informed using an unadjusted logistic regression analysis of pre‐operative Hb for the intervention strategy based on institution and procedure specific pre‐operative Hb. It is presented for each outcome (Hb < 70 g/L, Hb < 80 g/L, allogeneic blood transfusion) and each risk threshold (0.1%, 0.25%, 0.5%, 0.75%, and 1%).

##### Net reduction

We also calculated the test trade‐off, which is the net reduction in the number of unnecessary G&S and post‐operative blood tests (false positives) using procedure and institution specific pre‐operative Hb cut‐offs compared to the ‘Test all: intervention for all patients’ strategy. This number is equivalent to the number of avoided interventions, whilst keeping the number of true positives the same. For example, a net reduction of 0.04 means that, per 100 patients, four unnecessary G&S and post‐operative blood test interventions are avoided for the same level of necessary interventions. We also calculated and plotted the net reduction, over the risk thresholds.

Further details about the net benefit and net reduction calculations are provided in Supporting Information S1: Appendix A. The utility of testing patients according to risk of post‐operative anaemia was compared with all other intervention strategies for each outcome and procedure type.

#### 2.5.4 Additional analyses

We performed a sensitivity analysis using data from years 2015 to 2018 to account for any potential temporal changes in patient blood management and to reflect current practice. We also performed a sensitivity analysis for transfusions that took place prior to a formal postoperative Hb measurement which assumed that these patients had Hb < 70 g/L and Hb < 80 g/L before their transfusion. Missing data were described, and no imputation analysis was performed given the small amount of missing data. All analyses are complete case analyses.

## 3 RESULTS

**FIGURE 1:**
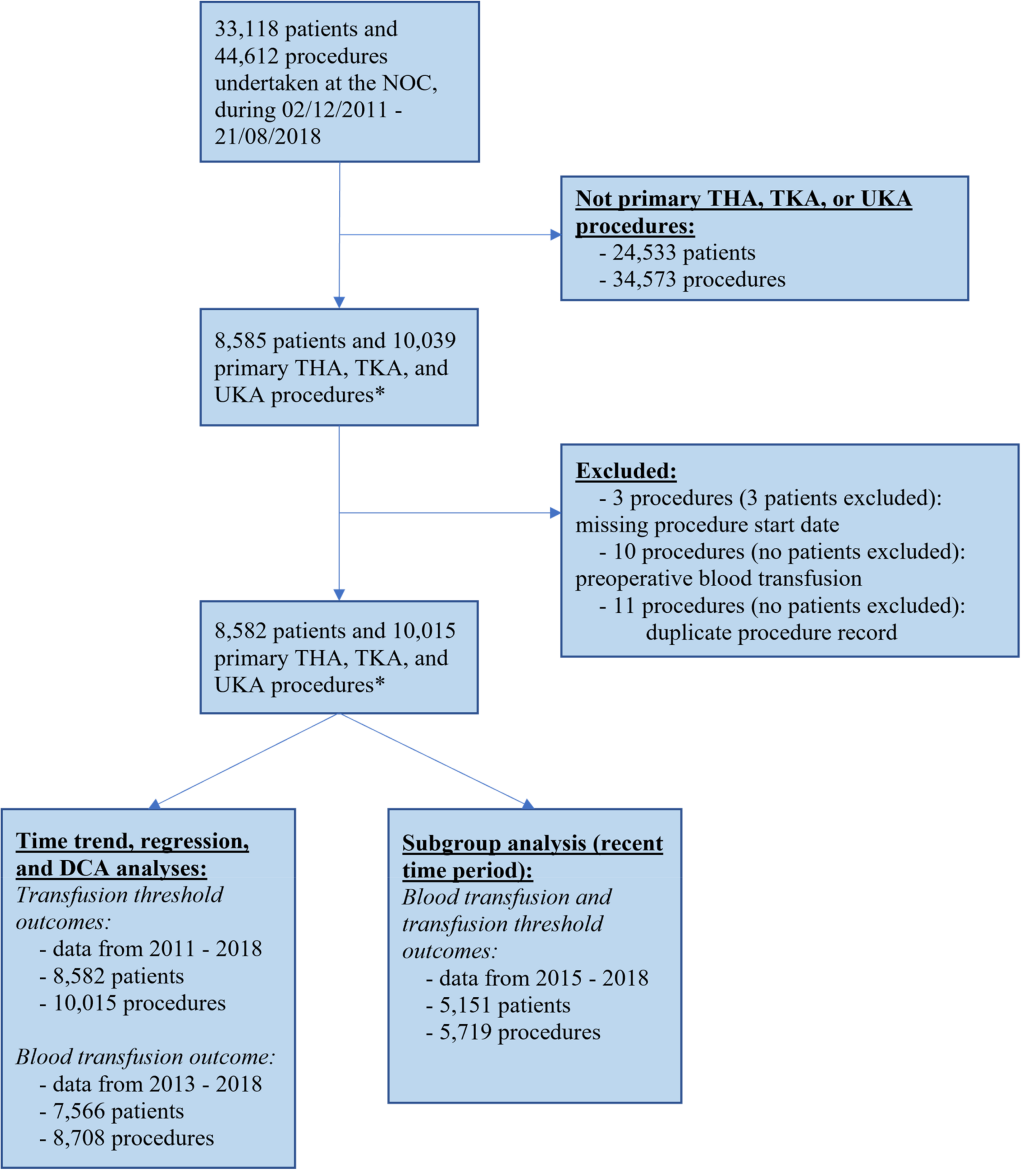
Flow of patients and procedures into the study. NOC, Nuffield Orthopaedic Centre, Oxford, UK; THA, total hip arthroplasty; TKA, total knee arthroplasty; UKA, unicompartmental (partial) knee arthroplasty; DCA, Decision curve analysis. *OPCS codes used to identify THA, TKA and UKA procedures: W371, W381, W391, W941, W421, W401, W581

44 612 surgical procedures were undertaken at the NOC between January 2011 and August 2018. Of these, 10 015 procedures were THA, TKA and UKA procedures (Figure 1).

### 3.1 Baseline and post‐operative characteristics

Of the 10 015 procedures included in the analysis, 49.1% were THA (*n* = 4917), 23.6% were TKA (*n* = 2363), and 27.3% were UKA (*n* = 2735). Patients had a mean age of 68 years (SD 11.7), 59.1% were female. Mean pre‐operative Hb was 138.8 g/L (SD 13.7) (Table 1), with 14.8% anaemic according to WHO criteria and 30.4% according to ICS criteria. 4.5% (*n* = 388/8708) patients received allogeneic blood, but only 3.9% (*n* = 15/388) of these patients had a post‐operative Hb below 70 g/L and 27.8% (*n* = 108/388) below 80 g/L prior to transfusion. 22.9% (*n* = 89/388) of blood transfusions were performed on the day of surgery, of which 12 were performed intraoperatively (13.5%, *n* = 12/89) (Table 2).

**TABLE 1:** Baseline characteristics of the study sample and procedures undertaken between 2011 and 2018, by procedure.

Baseline characteristics	THA (*n* = 4917)		TKA (*n* = 2363)		UKA (*n* = 2735)		Total (*n* = 10 015)	
	Mean	SD	Mean	SD	Mean	SD	Mean	SD
Age (years)	68.46	12.66	70.55	10.86	67.61	10.43	68.72	11.72
Pre‐operative Hb (g/L)	135.08	13.99	134.58	13.6	138.8	12.84	135.96	13.71
Missing (*n*, %)	39 (0.79)		110 (4.66)		125 (4.57)		274 (2.74)	
	***n***	**%**	***n***	**%**	***n***	**%**	***n***	**%**
Sex								
Female	3021	61.44	1469	62.17	1433	52.39	5923	59.14
Male	1896	38.56	894	37.83	1302	47.61	4092	40.86
ASA								
1	718	14.6	236	9.99	455	16.64	1409	14.07
2	2928	59.55	1585	67.08	1826	66.76	6339	63.3
3	943	19.18	427	18.07	314	11.48	1684	16.81
4	34	0.69	4	0.17	2	0.07	40	0.4
Missing	294	5.98	111	4.7	138	5.05	543	5.42
Pre‐operative anaemia (WHO)								
No	4062	82.61	1854	78.46	2340	85.56	8256	82.44
Yes	816	16.6	399	16.89	270	9.87	1485	14.83
Missing	39	0.79	110	4.66	125	4.57	274	2.74
Pre‐operative anaemia (ICS)								
No	3240	65.89	1469	62.17	1984	72.54	6693	66.83
Yes	1638	33.31	784	33.18	626	22.89	3048	30.43
Missing	39	0.79	110	4.66	125	4.57	274	2.74

Abbreviations: THA, total hip arthroplasty; TKA, total knee arthroplasty; UKA, unicompartmental (partial) knee arthroplasty; Hb, haemoglobin; ASA, American Society of Anesthesiologists; WHO, World Health Organisation; ICS, International Consensus Statement.

**TABLE 2:** Post‐operative characteristics of the study sample for procedures performed between 2011 and 2018 for transfusion threshold analyses and between 2013 and 2018 for the blood transfusion analysis.

Post‐operative characteristics	THA (*n* = 4917)		TKA (*n* = 2363)		UKA (*n* = 2735)		Total (*n* = 10 015)	
	Mean	SD	Mean	SD	Mean	SD	Mean	SD
Post‐operative Hb (g/L)	107.53	15.3	109.78	14.1	121.13	13.19	110.89	15.51
Missing (n, %)	100 (2.03)		117 (4.95)		908 (33.2)		1125 (11.23)	
Change in Hb (g/L)	27.52	11.25	24.74	10.47	17.16	8.03	24.71	11.21
Missing (*n*, %)	124 (2.52)		157 (6.64)		947 (34.63)		1228 (12.26)	
	***n***	**%**	***n***	**%**	***n***	**%**	***n***	**%**
Post‐operative anaemia (WHO)								
No	598	12.16	329	13.92	727	26.58	1654	16.52
Yes	4219	85.8	1917	81.13	1100	40.22	7236	72.25
Missing	100	2.03	117	4.95	908	33.2	1125	11.23
Post‐operative anaemia (ICS)								
No	348	7.08	161	6.81	448	16.38	957	9.56
Yes	4469	90.89	2085	88.24	1379	50.42	7933	79.21
Missing	100	2.03	117	4.95	908	33.2	1125	11.23
Post‐operative blood transfusion^a^								
No	3946	92.89	1955	95.93	2419	99.88	8320	95.54
Yes	302	7.11	83	4.07	3	0.12	388	4.46
Post‐operative blood transfusion given on same day of surgery^b^								
No	224	74.17	72	86.75	3	100	299	77.06
Yes	78	25.83	11	13.25	0	0	89	22.94
Post‐operative Hb below transfusion trigger (70 g/L)								
No	4794	97.5	2244	94.96	1827	66.8	8865	88.52
Yes	23	0.47	2	0.08	‐	‐	25	0.25
Missing	100	2.03	117	4.95	908	33.2	1125	11.23
Post‐operative Hb below transfusion trigger (80 g/L)								
No	4678	95.14	2216	93.78	1826	66.76	8720	87.07
Yes	139	2.83	30	1.27	1	0.04	170	1.70
Missing	100	2.03	117	4.95	908	33.2	1125	11.23

*Note*: Results are presented by procedure.

Abbreviations: THA, total hip arthroplasty; TKA, total knee arthroplasty; UKA, unicompartmental (partial) knee arthroplasty; Hb, haemoglobin; ASA, American Society of Anesthesiologists; WHO, World Health Organisation; ICS, International Consensus Statement.

^a^ Blood transfusion based on data from 2013 to 2018, *n* = 8708.

^b^ Patients who had a blood transfusion based on data from 2013 to 2018.

Thirty‐one blood transfusions were administered on the day of surgery but before a formal postoperative Hb was available and a further 17 blood transfusions were performed after the day of surgery but before a formal postoperative Hb was available. Of these 48 blood transfusions, two patients triggered the post‐surgery 70 g/L transfusion threshold despite having received a blood transfusion and nine triggered the post‐surgery 80 g/L transfusion threshold.

No patient developed a post‐operative Hb below 70 g/L, and one patient (0.04%) developed a post‐operative Hb below 80 g/L following UKA. Two patients (0.08%) developed a post‐operative Hb below 70 g/L following TKA. Regression and decision curve analysis was not performed for these procedure outcomes due to the small number of cases.

### 3.2 What are the temporal trends of post‐operative Hb below the 70 and 80 g/L transfusion thresholds, and allogeneic blood transfusion following elective primary hip and knee arthroplasty?

**FIGURE 2:**
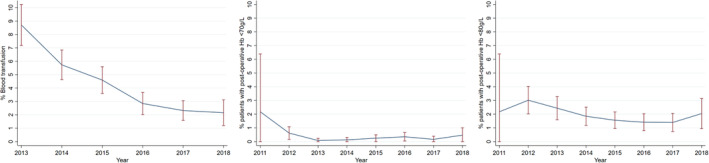
Time trends of observed blood transfusion (2013–2018), and post‐operative Hb less than 70 and 80 g/L transfusion thresholds (2011–2018) for all procedures.

The proportion of patients receiving blood transfusions fell during the period of study, although the proportion of patients developing post‐operative anaemia remained stable (Figure 2).

### 3.3 What is the relationship between pre‐operative Hb, and post‐operative Hb and blood transfusion?

Odds of patients developing a post‐operative Hb below 70 or 80 g/L, and patients receiving an allogeneic blood transfusion reduced per unit increase in pre‐operative Hb for THA and TKA procedures (Figure S1 and Table S2). After adjustment, the odds of receiving allogeneic blood were 7% (OR 0.93, 95% CI 0.92–0.94) lower after THA and 8% lower (OR 0.92, 95% CI 0.90–0.94) lower after TKA per unit (g/L) increase in pre‐operative Hb.

### 3.4 What are the pre‐operative Hb thresholds for different risks of severe post‐operative anaemia?

Pre‐operative Hb thresholds for different risks of post‐operative anaemia differed between THA and TKA (Table 3). An example scenario based on these results is a clinician who does not wish to unnecessarily collect blood for pre‐operative G&S in more than 99 patients to identify 1 patient with a post‐operative Hb less than the 80 g/L transfusion threshold (1% risk threshold). They would select a pre‐operative Hb < 136 g/L to decide whether to perform a pre‐operative G&S for THA. In our cohort, a threshold of <136 g/L would have meant 130 patients who subsequently required a blood transfusion had a pre‐operative G&S performed pre‐operatively, and eight patients who subsequently required a blood transfusion did not have a G&S performed preoperatively and would need one prior to transfusion. G&S from 2478 patients would have been collected but not required, and no G&S would have been collected from 2177 patients who did not require a blood transfusion.

**TABLE 3:** Risk associated pre‐operative Hb thresholds and decision curve analysis results for THA and TKA.

Procedure	Outcome	Risk thresholds (%)	Harm to benefit ratio	Associated Hb cut‐off (g/L)	No. true positives	No. false positives	No. true negatives	No. false negatives	NB per 1000 patients: Hb (g/L) vs. none	NR per 100 patients: Hb (g/L) vs. all
THA	Post‐operative Hb below transfusion trigger (70 g/L)	1	1:99	<116	16	434	4336	7	2.4	76
		0.75	1:133	<118	18	533	4237	5	2.9	74.6
		0.5	1:199	<122	19	817	3953	4	3.2	67.6
		0.25	1:399	<127	20	1334	3436	3	3.5	46.7
		0.1	1:999	<139	23	3003	1767	0	3.9	10.5
	Post‐operative Hb below transfusion trigger (80 g/L)	1	1:99	<136	130	2478	2177	8	22	30.1
		0.75	1:133	<138	132	2755	1900	6	23.2	23.1
		0.5	1:199	<143	135	3393	1262	3	24.7	16.5
		0.25	1:399	<149	137	3950	705	1	26.5	6.4
		0.1	1:999	<164	138	4576	79	0	27.9	5
	Post‐operative blood transfusion^a^	1	1:99	<153	292	3552	368	9	60.7	−12.4
		0.75	1:133	<156	297	3677	243	4	63.8	−6.8
		0.5	1:199	<162	298	3823	97	3	66.1	−11.6
		0.25	1:399	<169	300	3886	34	1	68.8	−8.6
		0.1	1:999	<185	301	3917	3	0	70.4	0.2
TKA	Post‐operative Hb below transfusion trigger (80 g/L)	1	1:99	<123	29	443	1733	1	11.1	74.1
		0.75	1:133	<125	29	538	1638	1	11.3	68.3
		0.5	1:199	<128	29	681	1495	1	11.6	58.7
		0.25	1:399	<132	29	922	1254	1	12.1	38.8
		0.1	1:999	<143	30	1588	588	0	13	39.1
	Post‐operative blood transfusion^a^	1	1:99	<144	77	1450	479	6	31.2	−3.5
		0.75	1:133	<147	80	1581	348	3	33.8	−2.4
		0.5	1:199	<152	82	1754	175	1	35.9	−9.7
		0.25	1:399	<159	83	1868	61	0	38.9	3
		0.1	1:999	<177	83	1929	0	0	40.3	0.3

Abbreviations: THA, total hip arthroplasty; TKA, total knee arthroplasty; Hb, haemoglobin; NB, net benefit; NR, net reduction.

^a^ Blood transfusion based on data from 2013 to 2018.

### 3.5 What is the utility of different strategies using pre‐operative Hb to select patients for pre‐operative G&S and post‐operative Hb measurement?

**FIGURE 3:**
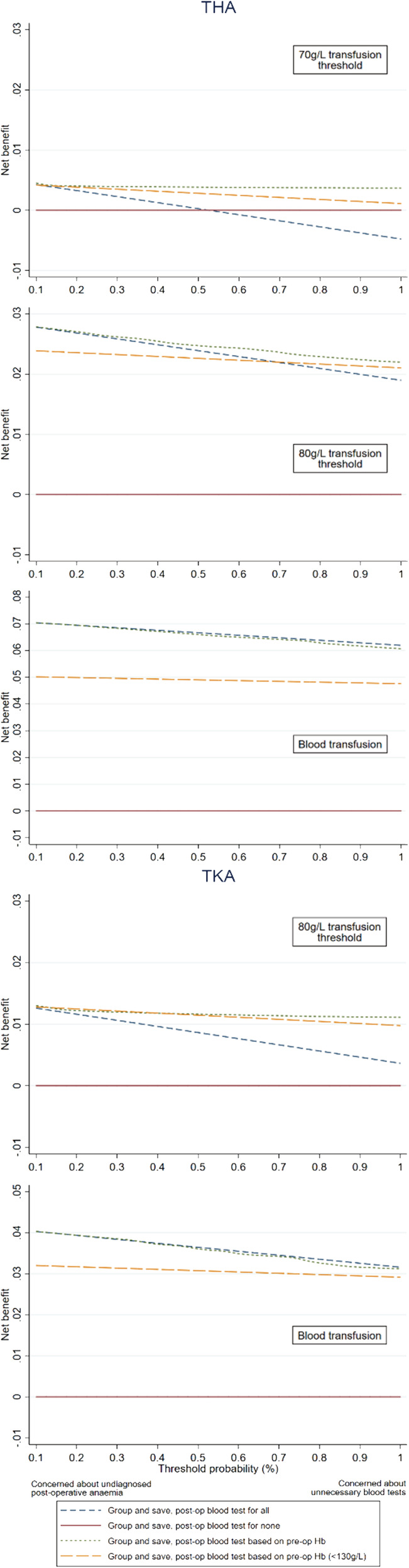
Decision curves for the four intervention strategies for THA and TKA, by outcome. The four intervention strategies are performing a G&S and post‐operative blood test on all patients, on no patients, only on patients with pre‐operative anaemia, or on patients at risk of post‐operative anaemia. Data are presented for 2011–2018 for transfusion threshold analyses, and 2013–2018 for the blood transfusion analysis. Analysis was not performed for a 70 g/L transfusion threshold for TKR due to the paucity of event rates. The *y*‐axis is the benefit (net benefit), and the x‐axis is the preference (threshold probability). The benefit of an intervention strategy is that is correctly identifies which patients had a post‐operative Hb < 70 g/L, <80 g/L or had a blood transfusion (and hence actually needed a G&S and postoperative blood test). The preference refers to how clinicians value different outcomes and what risk they are willing to take to not have blood when it is needed for a given patient, which varies. For example, if Clinician A was concerned about undiagnosed post‐operative anaemia in their THA (Hb < 70 g/L) patient, they may only be willing to accept a 0.1% risk to not have blood available when it is needed, compared to Clinician B who is more concerned about unnecessary blood test and would be willing to accept a slightly higher 1% risk of not having blood when it is needed. In this case for Clinician A, there is little difference between the intervention strategies of performing a G&S and post‐operative blood test on all patients, only on patients with pre‐operative anaemia, or on patients at risk of post‐operative anaemia. However, Clinician B would be better to use the intervention strategies of performing a G&S and post‐operative blood test only on patients at risk of post‐operative anaemia based on pre‐operative Hb measurement.

Decision curve analysis showed that for THA and TKA, using the ‘Test patients according to risk of post‐operative anaemia’ strategy resulted in improved net benefit across all risk thresholds for the 70 and 80 g/L transfusion threshold outcomes, when compared with the ‘Test none’ and ‘Test patients with pre‐operative anaemia’ strategies (Figure 3). A net reduction in unnecessary blood tests was found for these outcomes when using the ‘Test patients according to risk of post‐operative anaemia’ strategy, compared to the ‘Test all’ strategy (Figure S2 and Table S3).

Continuing the example scenario above, where a clinician selects a pre‐operative Hb threshold of 136 g/L for THA (corresponding to a 1% risk of post‐operative Hb < 80 g/L): Compared with a ‘test none’ approach, 22 additional patients per 1000 would be correctly selected for G&S without any unnecessary blood tests. Compared with a ‘test all’ approach, 30 additional patients per 100 would correctly have no G&S collected without decreasing the number of patients correctly selected for G&S.

Additional data of net benefit and net reduction comparisons between intervention strategies are provided in Figure S2 and Table S3. Only two patients undergoing TKR developed a post‐operative Hb below 70 g/L, and one patient undergoing UKR developed a post‐operative Hb below 80 g/L, hence decision curve analysis was not performed for these outcomes.

### 3.6 Sensitivity analysis

Thresholds for intervention were lower for TKA and THA using 2015–2018 compared with 2011–2018 data (Tables 3 and 4). Hb thresholds based on the ‘Test patients according to risk of post‐operative anaemia’ strategy demonstrated increased net benefit compared to the other strategies for the 70 and 80 g/L transfusion threshold outcomes (Figures S3 and S4, Table S4).

**TABLE 4:** Risk associated pre‐operative Hb thresholds and decision curve analysis results for THA and TKA using data limited to 2015–2018.

Procedure	Outcome	Risk thresholds (%)	Harm to benefit ratio	Associated Hb cut‐off (g/L)	No. true positives	No. false positives	No. true negatives	No. false negatives	NB per 1000 patients: Hb (g/L) vs. none	NR per 100 patients: Hb (g/L) vs. all
THA	Post‐operative Hb below transfusion trigger (70 g/L)	1	1:99	<116	12	240	2408	2	3.7	84
		0.75	1:133	<117	12	268	2380	2	3.8	79.5
		0.5	1:199	<120	12	359	2289	2	3.8	71.0
		0.25	1:399	<124	12	542	2106	2	4	49.1
		0.1	1:999	<134	13	1261	1387	1	4.5	26.2
	Post‐operative Hb below transfusion trigger (80 g/L)	1	1:99	<131	61	970	1627	4	19.1	45
		0.75	1:133	<133	61	1130	1467	4	19.7	35.2
		0.5	1:199	<137	62	1459	1138	3	20.7	22.9
		0.25	1:399	<141	65	1744	853	0	22.8	32.0
		0.1	1:999	<154	65	2385	212	0	23.6	16.6
	Post‐operative blood transfusion	1	1:99	<146	129	2024	530	11	40.3	−20.8
		0.75	1:133	<149	133	2162	392	7	43.3	−19.8
		0.5	1:199	<154	136	2342	212	4	45.8	−27.8
		0.25	1:399	<161	138	2484	70	2	48.9	−27.0
		0.1	1:999	<179	140	2549	5	0	51.0	0.5
TKA	Post‐operative Hb below transfusion trigger (80 g/L)	1	1:99	<120	8	177	1099	1	5.0	79.1
		0.75	1:133	<121	8	200	1076	1	5.1	73.4
		0.5	1:199	<125	8	304	972	1	5.1	62.0
		0.25	1:399	<128	8	394	882	1	5.5	37.6
		0.1	1:999	<139	9	794	482	0	5.8	−25.5
	Post‐operative blood transfusion	1	1:99	<139	32	786	489	3	18.7	17.5
		0.75	1:133	<142	34	891	384	1	20.8	19.2
		0.5	1:199	<147	34	1033	242	1	22.1	5.4
		0.25	1:399	<154	35	1180	95	0	24.5	7.3
		0.1	1:999	<170	0	3	0	0	25.8	1.3

Abbreviations: THA, total hip arthroplasty; TKA, total knee arthroplasty; Hb, haemoglobin; NB, net benefit; NR, net reduction.

Thresholds for intervention and the associated net benefit for using Hb thresholds based on the ‘Test patients according to risk of post‐operative anaemia’ strategy increased when THR patients who received a blood transfusion before their postoperative Hb was measured were assumed to have a Hb < 70 g/L. A smaller increase was found in the thresholds for intervention and associated net benefit when both THR and TKR patients, who received a blood transfusion before their postoperative Hb was measured were assumed to have a Hb < 80 g/L. Full results are provided in Table S5.

## 4 DISCUSSION

### 4.1 Summary of findings

Only a small proportion of patients have a post‐operative Hb less than 70 or 80 g/L following primary hip and knee arthroplasty. The odds of a post‐operative Hb less than 70 or 80 g/L or receiving a blood transfusion fall as pre‐operative Hb increases for both THA and TKA. We found the optimal strategy for selecting patients for pre‐operative G&S and post‐operative Hb measurement was to base the decision on a calculated risk of severe post‐operative anaemia. This strategy allows clinicians to select a harm to benefit ratio that is most appropriate for their clinical practice, and allows more accurate patient selection than when testing all or no patients, or patients with pre‐operative anaemia (Hb < 130 g/L). Although differences in net benefit are small between strategies, there is potential for considerable reduction in unnecessary G&S and post‐operative blood tests using a risk‐based approach. Only two patients undergoing TKR developed a post‐operative Hb below 70 g/L (0.09%) and one patient undergoing UKR developed a post‐operative Hb below 80 g/L (0.05%). In the 2015–2018 data, there was a 1% risk of post‐operative Hb < 80 g/L if pre‐operative Hb was <120 g/L for TKA and <131 g/L for THA.

There has been a reduction in the proportion of patients receiving a blood transfusion with time, likely due to improved blood management and restrictive transfusion strategies. However, the proportion of patients receiving a post‐operative blood transfusion remains higher than recommended by national guidelines, with most transfusions administered with a Hb >70 g/L. This observation highlights the need to promote adherence to recommended transfusion thresholds and avoid unnecessary transfusions and related complications.

### 4.2 Literature

Routinely performing blood tests for pre‐operative G&S and post‐operative Hb measurement is potentially unnecessary given the low prevalence of severe post‐operative anaemia and blood transfusion.^21^ When unnecessary, these blood tests increase cost and impair patient experience through venepuncture. They may also increase pre‐operative hospital attendance delay surgery (awaiting G&S), or discharge from hospital (awaiting post‐operative Hb). Previous studies with smaller sample sizes proposed thresholds for performing these investigations at 130 g/L or between 121 and 124 g/L depending on age.^12^, ^22^ The ROC analysis used in these studies have limited clinical interpretability and assumes true and false positives are equally important, which may not be accurate. When selecting patients for G&S, a true positive potentially has a higher misclassification cost and carry greater importance than a false positive.^23^

The ability to stratify risk using decision curve analysis, enables the selection of a threshold for G&S and post‐operative Hb measurement that is deemed appropriate for a specific clinical setting. These thresholds can be used for electronic clinical decision support tools increasingly adopted for preoperative assessment. In addition to the patient population and clinical practice, institutional factors may influence selection of appropriate harm to benefit ratios, such as the availability of electronic blood issue and proximity to blood banks. Clinicians must also assess the risk discharging patients with severe undiagnosed anaemia, and a different harm to benefit ratio Hb threshold may be used to select patients for G&S than for post‐operative Hb measurement. Rates of blood transfusion are falling and suggested thresholds for performing pre‐operative G&S and post‐operative Hb measurement require regular review.^21^

In this study, less than a quarter of transfusions were administered on the day of surgery, hence in most cases there is time to organise a G&S if not already performed. This can be expedited if there is a strong clinical suspicion that a transfusion will be unexpectedly required, such as higher than anticipated on table blood loss or post‐operative cardiovascular compromise. If cross‐matched blood products are not available in an emergency, the use of blood group O blood will be necessary. A circumstance when a group and save is always indicated is when there is a past medical history of red blood cell antibodies.

### 4.3 Strengths and limitations

Our study uses contemporary and clinically relevant statistical methodology to evaluate the utility of different strategies to select patients for pre‐operative G&S and post‐operative Hb measurement. We build on existing ROC curve analyses that assume true and false positives are equally important^24^, ^25^ and use decision curve analysis which better addresses the utility of a prognostic test. Decision curve analysis weights true and false positives separately, and does not require categorisation of pre‐operative Hb to derive clinically informed risk thresholds, which minimises the loss of statistical information.^26^ We present the results of decision curve analysis for different levels of risk that clinicians may find acceptable.

Our study is limited as we used data from a single centre and only collected data up to 2018 as the study observations have since been used to reduce the number of blood tests performed. No formal sample size calculation was performed. We included all procedures available from when this centre became fully ‘paperless’ and the sample size was informed by a relevant time interval. We believe any optimism in our estimates are negligible as we use a single continuous predictor for our predictions. Using recent guidance, we estimated that a minimum of 995 patients with five events (events per predictors = 4.68) would be needed to model risk in the THA analyses, based on achieving a conservative 15% of the maximum r‐squared and a 0.47% event rate (lowest event rate was found for the 70 g/L outcome).^27^ A minimum of 457 patients with six events (events per predictors = 5.80) is required for the TKA analyses, based on achieving a conservative 15% of the maximum *r*‐squared using one predictor and a 1.27% event rate (lowest event rate was found for the 80 g/L outcome). The heuristic shrinkage for each analysis ranged from 0.97 to 0.99, indicating likely very small amounts of optimism in our estimates. Missing data was described in our study but was not imputed. Given the very small amount of missing data present, a complete case analysis was justified.^28^ We used an unadjusted logistic regression model to inform the pre‐operative Hb thresholds for different risk of severe post‐operative anaemia strategy, instead of developing a prediction model for each outcome. However, the purpose of this study was to evaluate the individual effect of pre‐operative Hb and identify pre‐operative Hb cut‐off values that can easily be used in clinical practice when preparing to THA and TKA.

Our analysis includes 48 patients and procedures where a blood transfusion was performed prior to the availability of a formal postoperative Hb. Possible explanations are estimated blood loss and physiological parameters as an indication for blood loss or point of care Hb measurement. This observation may bias our results as the number of patients with a Hb lower than 70 or 80 g/L after surgery may be higher than what is reported. A sensitivity analysis assuming a postoperative Hb < 70 g/L or Hb < 80 g/L in this cohort showed an increase in the transfusion thresholds. We also accounted for this limitation by including postoperative transfusion as a secondary outcome measure, independent of postoperative Hb.

### 4.4 Future research

This study was performed on data from a single institution, but the methodology can be applied to different surgical procedures and different institutions. Clinicians overseeing the care of patients receiving primary hip and knee arthroplasty may wish to select an acceptable harm to benefit ratio Hb threshold from our study and then retrospectively validate the performance using their local data. There may be significant financial savings associated with reducing the number of unnecessary G&S and post‐operative blood tests, and a formal health economic review may be of value in the future.

### 4.5 Conclusions

In summary, our study outlines a means of selecting patients for G&S and post‐operative Hb measurement based on the risk of developing severe post‐operative anaemia. Pre‐operative G&S and post‐operative Hb measurement may not be indicated for UKA or TKA when adopting restrictive transfusion thresholds provided clinicians accept a 0.1% risk that patients will develop severe undiagnosed post‐operative anaemia (Hb < 70 g/L). The decision to perform these blood tests for THA patients should be based on local institutional data and selection of an acceptable harm to benefit ratio. Decision curve analysis can be adopted for all surgical procedures to accurately select patients who require pre‐operative G&S and post‐operative Hb measurement, facilitating day‐case surgery and reduced cost.

## Supporting information


**Appendix S1**: Supporting information.
