# Supplementary material for: Utility of pre‐operative haemoglobin concentration to guide peri‐operative blood tests for hip and knee arthroplasty: A decision curve analysis
Source: Transfus Med. 2022 May 11;32(4):306–17. doi: 10.1111/tme.12873 (PMC9541407; doi:10.1111/tme.12873)
Supplement: Supplementary file 1 — Appendix S1: Supporting information. [file TME-32-306-s001.docx]

**Supplementary Data**

**Supplementary table 1**. OPCS procedure codes for primary total hip and knee arthroplasty, and unicompartmental knee arthroplasty.

| **Procedure** | **OPCS code** | **OPCS description** |
| --- | --- | --- |
| Primary total hip arthroplasty (THA) | W371 | Primary total prosthetic replacement of hip joint using cement |
|  | W381 | Primary total prosthetic replacement of hip joint not using cement |
|  | W391 | Primary total prosthetic replacement of hip joint NEC |
|  | W941 | Primary hybrid prosthetic replacement of hip joint using cemented femoral component |
| Primary total knee arthroplasty (TKA) | W401 | Primary total prosthetic replacement of knee joint using cement |
|  | W421 | Primary total prosthetic replacement of knee joint NEC |
| Unicompartmental knee arthroplasty (UKA) | W581 | Primary resurfacing arthroplasty of joint |

**Appendix A: Decision Curve Analysis methods**

**Net benefit**

Net benefit corrects the proportion of true positives identified by the intervention strategy for the number of false positives. The lower the desired risk threshold, the lower the perceived harm of false positives. Therefore, the amount by which net benefit corrects for false positives is determined by the exchange rate, where N is the sample size (Eq.1):

| $Net benefit= \frac{true positives}{N}-\left( \frac{false positives}{N}\times\frac{1}{exchange rate} \right)$, | Eq.1 |
| --- | --- |

The net benefit is the net proportion of true positives, much like net profit equals revenue minus all expenditures in business. Net benefit is equivalent to the proportion of true positives in the absence of false positives (perfect specificity). For example, a net benefit of 0.002 is equivalent to having 2 patients being correctly identified as needing blood transfusion per 1000 patients, without incorrectly selecting anyone who does not need a blood transfusion. The net benefit is directly comparable to the *‘test none’* strategy (do not perform pre-operative G&S or post-operative Hb measurement for anyone) which by definition has a net benefit of 0 because if you never intervene you do not have any true or false positives. The difference in net benefit between all strategies can also be calculated, for example if the net benefit increases from 0.001 when using ‘*Test all: intervention for all patients’* to 0.002 when using the ‘*Test patients according to risk of post-operative anaemia*’ strategy, the latter has 1 more net detected blood transfusion per 1000 patients for the same number of unnecessary G&S and post-operative blood test carried out.

The net benefit was calculated and presented in a decision curve at risk thresholds between 0.1% and 1%, as it was deemed unlikely a clinician would risk not having blood available when the risk of needing it exceeds 1% . The DCA is informed using an unadjusted logistic regression analysis of pre-operative Hb for the intervention strategy based on institution and procedure specific pre-operative Hb, and is presented for each outcome (Hb<70g/L, Hb<80g/L, allogeneic blood transfusion), and each risk threshold (0.1%, 0.25%, 0.5%, 0.75%, and 1%).

**Net reduction/Test trade-off**

We calculated the test trade-off, net reduction in the number of unnecessary G&S and post-operative blood tests (false positives) using procedure and institution specific pre-operative Hb cut-offs compared to the ‘*Test all: intervention for all patients’* strategy. This number is equivalent to the number of avoided interventions, whilst keeping the number of true positives the same. For example, a net reduction of 0.04 means that, per 100 patients, 4 unnecessary G&S and post-operative blood test interventions are avoided for the same level of necessary interventions.

The net reduction was calculated using the change in net benefit between the intervention strategies at each risk threshold (*p_t_*) (Eq.2).

| $Net reduction \left( avoidable interventions \right)=\frac{\left( {net benefit}_{pre-operative Hb}-{net benefit}_{all} \right)}{\left( \frac{p_{t}}{1-p_{t}} \right)}$ | Eq.2 |
| --- | --- |

**Supplementary table 2**. Unadjusted and adjusted logistic regression for pre-operative Hb (g/L) and patients having a post-operative Hb less than the transfusion thresholds (70g/L and 80g/L) and post-operative blood transfusion. Values are total number analysed (n), number of events (events), odds ratio (OR), and 95% confidence interval (CI). Analysis was stratified by procedure. Values were adjusted for age, sex and ASA, unless otherwise stated. Data between 2011 and 2018 was used for transfusion threshold analyses and between 2013 and 2018 for the blood transfusion analysis.

|  | | | **Outcomes** | | | | | | | | | | | | |
| --- | --- | --- | --- | --- | --- | --- | --- | --- | --- | --- | --- | --- | --- | --- | --- |
|  |  |  | **Post-operative Hb below transfusion trigger (70g/L)** | | | | | **Post-operative Hb below transfusion trigger (80g/L)** | | | | **Post-operative blood transfusion*** | | | |
|  |  |  | **events/n** | | **OR** | **95% CI** | **p-value** | **events/n** | **OR** | **95% CI** | **p-value** | **events/n** | **OR** | **95% CI** | **p-value** |
| **Pre-operative Hb g/L** | THA – Unadjusted | 23/4793 | | 0.88 | | (0.85-0.91) | <0.001 | 138/4793 | 0.90 | (0.89-0.91) | <0.001 | 320/4878 | 0.92 | (0.91-0.93) | <0.001 |
|  | THA – Adjusted | 20/4562 | | 0.87 | | (0.83-0.91) | <0.001 | 125/4562 | 0.89 | (0.88-0.91) | <0.001 | 291/4092 | 0.93 | (0.92-0.94) | <0.001 |
|  | TKA – Unadjusted | 2/2206 | | - | | - | - | 30/2206 | 0.86 | (0.83-0.89) | <0.001 | 91/2253 | 0.91 | (0.90-0.93) | <0.001 |
|  | TKA – Adjusted | 2/2206 | | - | | - | - | 30/2177 | 0.86 | (0.83-0.89) | <0.001 | 82/1980 | 0.92 | (0.90-0.94) | <0.001 |
|  | UKA – Unadjusted | 0/1827 | | - | | - | - | 1/1788 | - | - | - | 4/2610 | - | - | - |
|  | UKA – Adjusted | 0/1827 | | - | | - | - | 1/1788 | - | - | - | 3/2323 | - | - | - |

*using data from 2013-2018

THA, total hip arthoplasty; TKA, total knee arthroplasty; UKA, unicompartmental (partial) knee arthroplasty; Hb, haemoglobin; ASA, American Society of Anesthesiologists


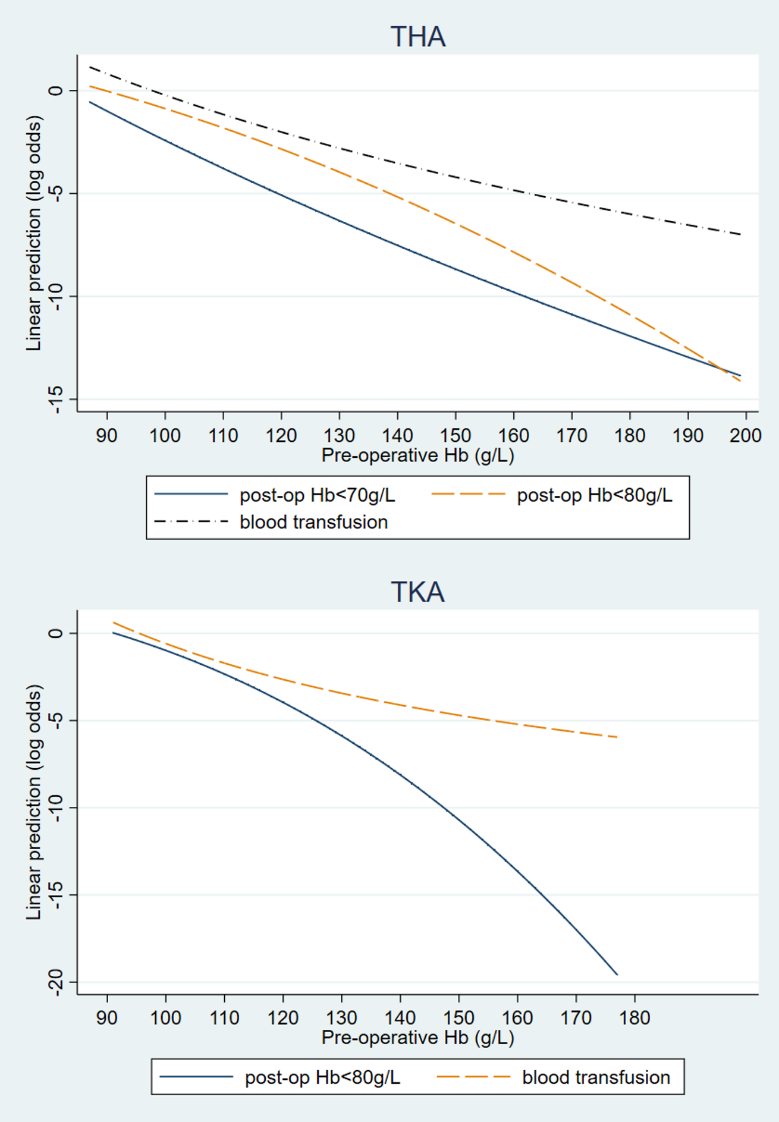


**Supplementary figure 1**. Fractional polynomials show that the linearity assumption of the relationship between pre-operative Hb and each outcome is satisfied for THA and TKA.

**Supplementary table 3** Risk associated pre-operative Hb thresholds and Decision Curve Analysis results for THA and TKA

| **Procedure** | **Outcome** | **Risk threshold** | **Harm to benefit ratio** | **Pre-operative Hb g/L** | | | | | | | **NB, per 1000 patients: intervention for all vs none** | **NB, per 1000 patients: Hb (<130g/L) vs none** |
| --- | --- | --- | --- | --- | --- | --- | --- | --- | --- | --- | --- | --- |
|  |  |  |  | **Associated Hb cut-off (g/L)** | **No. true positives** | **No. false positives** | **No. true negatives** | **No. false negatives** | **NB, per 1000 patients: using Hb (g/L) vs. none** | **NR per 100 patients: Hb (g/L) vs. all** |  |  |
| **THA** | Post-operative Hb below transfusion trigger (70g/L) | 1 | 1:99 | <116 | 16 | 434 | 4336 | 7 | 2.4 | 76.0 | -5.3 | 0.8 |
|  |  | 0.75 | 1:133 | <118 | 18 | 533 | 4237 | 5 | 2.9 | 74.6 | -2.7 | 1.7 |
|  |  | 0.5 | 1:199 | <122 | 19 | 817 | 3953 | 4 | 3.2 | 67.6 | -0.2 | 2.5 |
|  |  | 0.25 | 1:399 | <127 | 20 | 1334 | 3436 | 3 | 3.5 | 46.7 | 2.3 | 3.3 |
|  |  | 0.1 | 1:999 | <139 | 23 | 3003 | 1767 | 0 | 3.9 | 10.5 | 3.8 | 3.8 |
|  | Post-operative Hb below transfusion trigger (80g/L) | 1 | 1:99 | <136 | 130 | 2478 | 2177 | 8 | 22.0 | 30.1 | 19.0 | 21.0 |
|  |  | 0.75 | 1:133 | <138 | 132 | 2755 | 1900 | 6 | 23.2 | 23.1 | 21.5 | 21.8 |
|  |  | 0.5 | 1:199 | <143 | 135 | 3393 | 1262 | 3 | 24.7 | 16.5 | 23.9 | 22.6 |
|  |  | 0.25 | 1:399 | <149 | 137 | 3950 | 705 | 1 | 26.5 | 6.4 | 26.4 | 23.4 |
|  |  | 0.1 | 1:999 | <164 | 138 | 4576 | 79 | 0 | 27.9 | 5.0 | 27.8 | 23.9 |
|  | Post-operative blood transfusion* | 1 | 1:99 | <153 | 292 | 3552 | 368 | 9 | 60.7 | -12.4 | 61.9 | 47.6 |
|  |  | 0.75 | 1:133 | <156 | 297 | 3677 | 243 | 4 | 63.8 | -6.8 | 64.3 | 48.3 |
|  |  | 0.5 | 1:199 | <162 | 298 | 3823 | 97 | 3 | 66.1 | -11.6 | 66.6 | 49.0 |
|  |  | 0.25 | 1:399 | <169 | 300 | 3886 | 34 | 1 | 68.8 | -8.6 | 69.0 | 49.7 |
|  |  | 0.1 | 1:999 | <185 | 301 | 3917 | 3 | 0 | 70.4 | 0.2 | 70.4 | 50.2 |
| **TKA** | Post-operative Hb below transfusion trigger (80g/L) | 1 | 1:99 | <123 | 29 | 443 | 1733 | 1 | 11.1 | 74.1 | 3.6 | 9.8 |
|  |  | 0.75 | 1:133 | <125 | 29 | 538 | 1638 | 1 | 11.3 | 68.3 | 6.1 | 10.6 |
|  |  | 0.5 | 1:199 | <128 | 29 | 681 | 1495 | 1 | 11.6 | 58.7 | 8.6 | 11.5 |
|  |  | 0.25 | 1:399 | <132 | 29 | 922 | 1254 | 1 | 12.1 | 38.8 | 11.1 | 12.3 |
|  |  | 0.1 | 1:999 | <143 | 30 | 1588 | 588 | 0 | 13.0 | 39.1 | 12.6 | 12.8 |
|  | Post-operative blood transfusion* | 1 | 1:99 | <144 | 77 | 1450 | 479 | 6 | 31.2 | -3.5 | 31.6 | 29.1 |
|  |  | 0.75 | 1:133 | <147 | 80 | 1581 | 348 | 3 | 33.8 | -2.4 | 34.0 | 29.9 |
|  |  | 0.5 | 1:199 | <152 | 82 | 1754 | 175 | 1 | 35.9 | -9.7 | 36.4 | 30.7 |
|  |  | 0.25 | 1:399 | <159 | 83 | 1868 | 61 | 0 | 38.9 | 3.0 | 38.8 | 31.5 |
|  |  | 0.1 | 1:999 | <177 | 83 | 1929 | 0 | 0 | 40.3 | 0.3 | 40.3 | 32.0 |

*Blood transfusion based on data from 2013-2018

THA, total hip arthroplasty; TKA, total knee arthroplasty; Hb, haemoglobin; NB, net benefit; NR, net reduction.


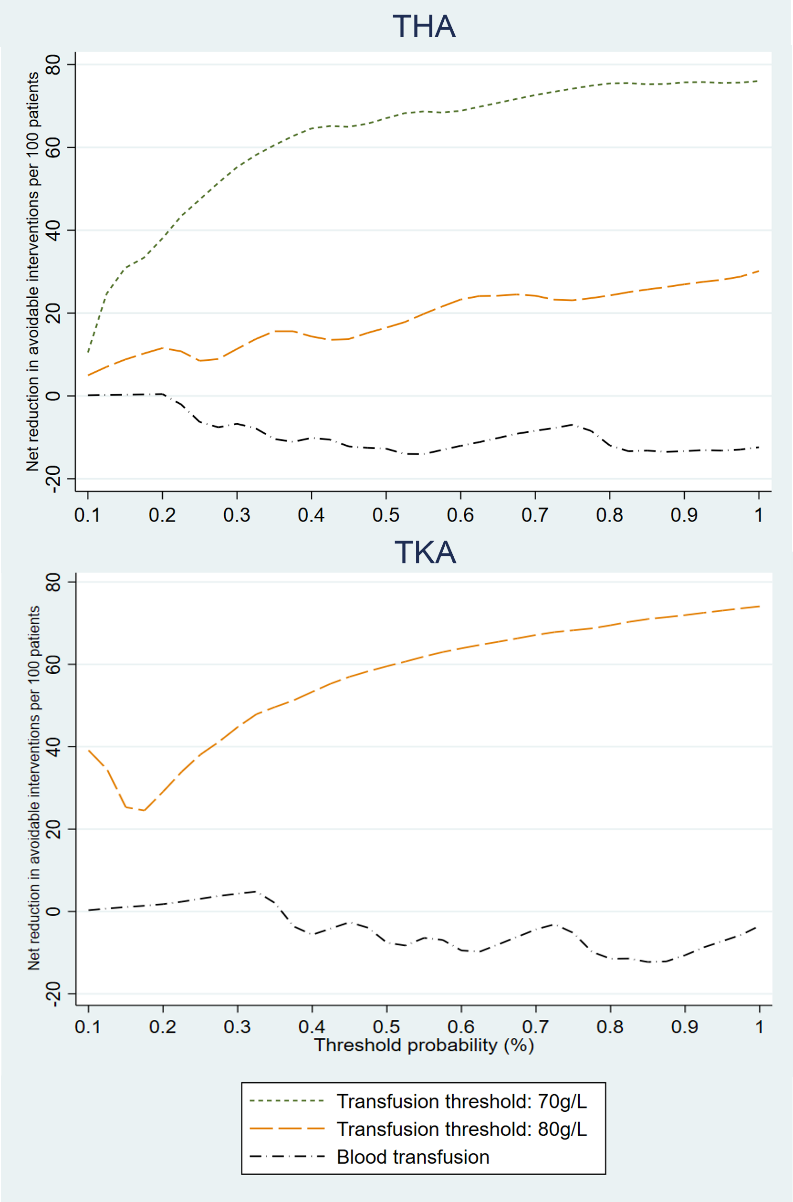


**Supplementary figure 2**. Net reduction in unnecessary G&S and post-operative blood tests using continuous pre-operative Hb compared to *‘**Test all: intervention for all patients*’ strategy, to inform selection of patient at risk of each outcome for THA and TKA. Data is presented for 2011-2018 for transfusion threshold analyses, and 2013-2018 for the blood transfusion analysis.

**Supplementary table 4** Risk associated pre-operative Hb thresholds and Decision Curve Analysis results for THA and TKA using data limited to 2015-2018

| **Procedure** | **Outcome** | **Risk threshold** | **Harm to benefit ratio** | **Pre-operative Hb g/L** | | | | | | | **NB, per 1000 patients: intervention for all vs none** | **NB, per 1000 patients: Hb (<130g/L) vs none** |
| --- | --- | --- | --- | --- | --- | --- | --- | --- | --- | --- | --- | --- |
|  |  |  |  | **Associated Hb cut-off (g/L)** | **No. true positives** | **No. false positives** | **No. true negatives** | **No. false negatives** | **NB, per 1000 patients: using Hb (g/L) vs. none** | **NR per 100 patients: Hb (g/L) vs. all** |  |  |
| **THA** | Post-operative Hb below transfusion trigger (70g/L) | 1 | 1:99 | <116 | 12 | 240 | 2408 | 2 | 3.70 | 84.00 | -4.79 | 1.11 |
|  |  | 0.75 | 1:133 | <117 | 12 | 268 | 2380 | 2 | 3.75 | 79.46 | -2.26 | 1.96 |
|  |  | 0.5 | 1:199 | <120 | 12 | 359 | 2289 | 2 | 3.83 | 71.04 | 0.26 | 2.82 |
|  |  | 0.25 | 1:399 | <124 | 12 | 542 | 2106 | 2 | 4.00 | 49.14 | 2.77 | 3.66 |
|  |  | 0.1 | 1:999 | <134 | 13 | 1261 | 1387 | 1 | 4.53 | 26.22 | 4.26 | 4.17 |
|  | Post-operative Hb below transfusion trigger (80g/L) | 1 | 1:99 | <131 | 61 | 970 | 1627 | 4 | 19.11 | 45.00 | 14.56 | 18.94 |
|  |  | 0.75 | 1:133 | <133 | 61 | 1130 | 1467 | 4 | 19.71 | 35.22 | 17.05 | 19.75 |
|  |  | 0.5 | 1:199 | <137 | 62 | 1459 | 1138 | 3 | 20.66 | 22.88 | 19.52 | 20.56 |
|  |  | 0.25 | 1:399 | <141 | 65 | 1744 | 853 | 0 | 22.78 | 32.04 | 21.97 | 21.36 |
|  |  | 0.1 | 1:999 | <154 | 65 | 2385 | 212 | 0 | 23.61 | 16.60 | 23.44 | 21.84 |
|  | Post-operative blood transfusion* | 1 | 1:99 | <146 | 129 | 2024 | 530 | 11 | 40.30 | -20.75 | 42.39 | 37.43 |
|  |  | 0.75 | 1:133 | <149 | 133 | 2162 | 392 | 7 | 43.30 | -19.83 | 44.80 | 38.19 |
|  |  | 0.5 | 1:199 | <154 | 136 | 2342 | 212 | 4 | 45.80 | -27.84 | 47.20 | 38.95 |
|  |  | 0.25 | 1:399 | <161 | 138 | 2484 | 70 | 2 | 48.91 | -27.02 | 49.59 | 39.71 |
|  |  | 0.1 | 1:999 | <179 | 140 | 2549 | 5 | 0 | 51.02 | 0.52 | 51.02 | 40.16 |
| **TKA** | Post-operative Hb below transfusion trigger (80g/L) | 1 | 1:99 | <120 | 8 | 177 | 1099 | 1 | 4.96 | 79.07 | -3.03 | 2.92 |
|  |  | 0.75 | 1:133 | <121 | 8 | 200 | 1076 | 1 | 5.05 | 73.44 | -0.50 | 3.75 |
|  |  | 0.5 | 1:199 | <125 | 8 | 304 | 972 | 1 | 5.13 | 61.95 | 2.01 | 4.58 |
|  |  | 0.25 | 1:399 | <128 | 8 | 394 | 882 | 1 | 5.46 | 37.59 | 4.52 | 5.40 |
|  |  | 0.1 | 1:999 | <139 | 9 | 794 | 482 | 0 | 5.76 | -25.45 | 6.01 | 5.90 |
|  | Post-operative blood transfusion* | 1 | 1:99 | <139 | 32 | 786 | 489 | 3 | 18.65 | 17.48 | 16.89 | 17.44 |
|  |  | 0.75 | 1:133 | <142 | 34 | 891 | 384 | 1 | 20.81 | 19.21 | 19.36 | 18.24 |
|  |  | 0.5 | 1:199 | <147 | 34 | 1033 | 242 | 1 | 22.10 | 5.42 | 21.83 | 19.03 |
|  |  | 0.25 | 1:399 | <154 | 35 | 1180 | 95 | 0 | 24.46 | 7.25 | 24.28 | 19.82 |
|  |  | 0.1 | 1:999 | <170 | 0 | 3 | 0 | 0 | 25.76 | 1.30 | 25.74 | 20.30 |

THA, total hip arthroplasty; TKA, total knee arthroplasty; Hb, haemoglobin; NB, net benefit; NR, net reduction.


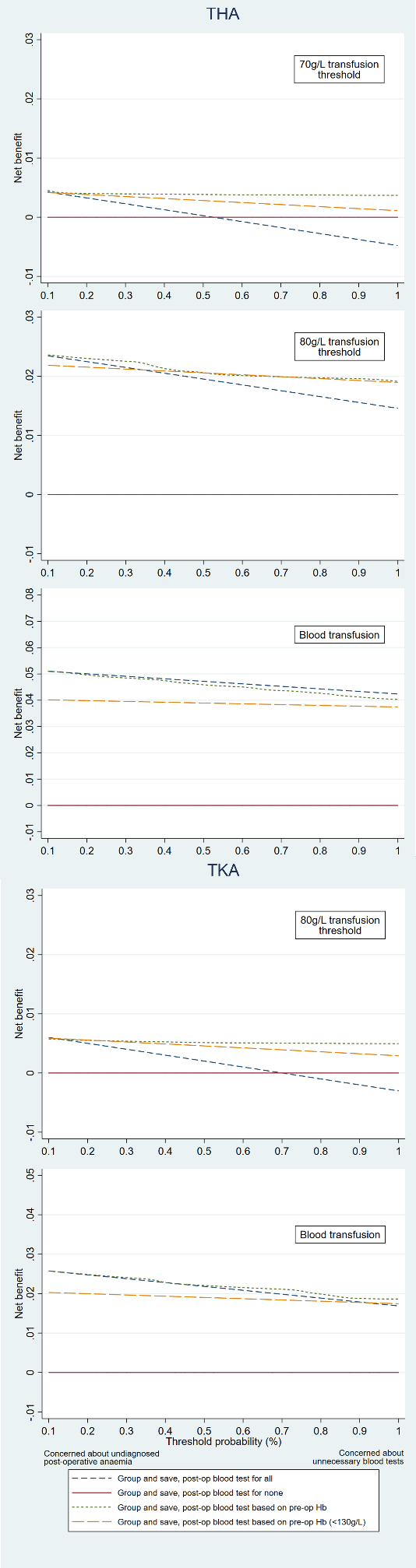


**Supplementary figure 3:** Decision curves for the four intervention strategies for THA and TKA, by outcome. Data is presented for 2015-2018.

**
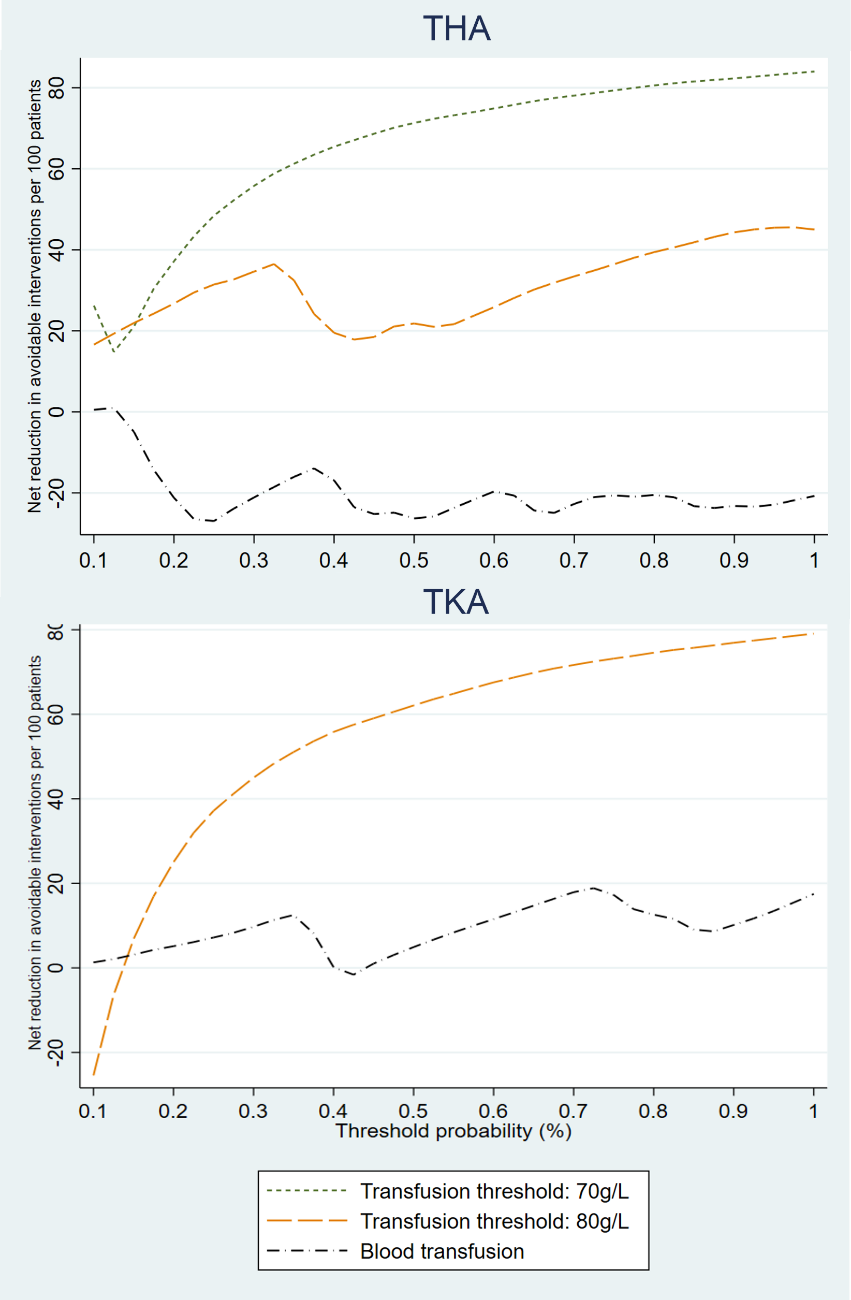
**

**Supplementary figure 4** Net reduction in unnecessary interventions using continuous pre-operative Hb for each outcome (2015-2018).

**Supplementary table 5** Risk associated pre-operative Hb thresholds and Decision Curve Analysis results for THA and TKA assuming patients where blood transfusion is given before postoperative Hb is taken had Hb<70g/L or 80g/L in the respective analysis. Analysis is restricted to 2013-2018.

| **Procedure** | **Outcome** | **Risk thresholds (%)** | **Harm to benefit ratio** | **Associated Hb cut-off (g/L)** | **No. true positives** | **No. false positives** | **No. true negatives** | **No. false negatives** | **NB per 1000 patients: Hb (g/L) vs. none** | **NR per 100 patients: Hb (g/L) vs. all** |
| --- | --- | --- | --- | --- | --- | --- | --- | --- | --- | --- |
| **THA** | Post-operative Hb below transfusion trigger (70g/L) | 1 | 1:99 | <127 | 46 | 1120 | 2972 | 11 | 8.4 | 45.4 |
|  |  | 0.75 | 1:133 | <129 | 48 | 1346 | 2746 | 9 | 9.1 | 37.5 |
|  |  | 0.5 | 1:199 | <134 | 50 | 1932 | 2160 | 7 | 9.9 | 21.5 |
|  |  | 0.25 | 1:399 | <139 | 52 | 2554 | 1538 | 5 | 11.0 | -11.0 |
|  |  | 0.1 | 1:999 | <154 | 55 | 3775 | 317 | 2 | 12.2 | -53.8 |
|  | Post-operative Hb below transfusion trigger (80g/L) | 1 | 1:99 | <138 | 137 | 2349 | 1654 | 9 | 27.4 | 19.0 |
|  |  | 0.75 | 1:133 | <140 | 140 | 2575 | 1428 | 6 | 29.1 | 15.3 |
|  |  | 0.5 | 1:199 | <145 | 141 | 3085 | 918 | 5 | 30.3 | -0.1 |
|  |  | 0.25 | 1:399 | <150 | 144 | 3471 | 532 | 2 | 32.6 | -6.4 |
|  |  | 0.1 | 1:999 | <165 | 146 | 3943 | 60 | 0 | 34.0 | -20.0 |
| **TKA** | Post-operative Hb below transfusion trigger (80g/L) | 1 | 1:99 | <126 | 22 | 518 | 1425 | 4 | 8.7 | 54.4 |
|  |  | 0.75 | 1:133 | <128 | 22 | 608 | 1335 | 4 | 8.8 | 40.9 |
|  |  | 0.5 | 1:199 | <132 | 24 | 821 | 1122 | 2 | 10.2 | 39.7 |
|  |  | 0.25 | 1:399 | <136 | 25 | 1055 | 888 | 1 | 11.4 | 24.8 |
|  |  | 0.1 | 1:999 | <149 | 26 | 1675 | 268 | 0 | 12.5 | 26.3 |

*Blood transfusion based on data from 2013-2018.

THA, total hip arthroplasty; TKA, total knee arthroplasty; Hb, haemoglobin; NB, net benefit; NR, net reduction.
